# Animal studies on the modulation of differential efficacy of polyethylene glycol loxenatide by intestinal flora

**DOI:** 10.3389/fendo.2025.1508473

**Published:** 2025-06-19

**Authors:** Dang Wenjiao, Wang Yurou, Xie Jiaqi, He Yan, Ji Hongfang, Chen Min, Guo Jianjin

**Affiliations:** Department of Geriatric Medicine, Shanxi Bethune Hospital, Shanxi Academy of Medical Sciences, Third Hospital of Shanxi Medical University, Tongji Shanxi Hospital, Taiyuan, China

**Keywords:** type 2 diabetes, intestinal flora, GLP-1 receptor agonists, polyethylene glycol exenatide, fecal microbiota transplantation, 16S rDNA

## Abstract

**Background:**

Gut microbiota has demonstrated an increasingly important role in the onset and development of type 2 diabetes mellitus (T2DM), Further investigations have revealed the interactions between drugs and the gut microbiome. However, there are still gaps in research regarding the potential interactions between the gut microbiota and GLP-1 and their therapeutic response in people with T2DM. In addition, Fecal microbiota transplantation (FMT) has become a promising strategy for patients with T2DM.

**Design, animals and measurements:**

50 healthy male C57BL/6 mice were fed a high-fat diet in combination with STZ to establish a T2DM mouse model. 40 mice were divided into the T2DM group (n=10) and the PEX168 group (n=30). the PEX168 group was divided into two subgroups of the IE group (HbA1c ≤6. 5%, n=12) and the SE group (HbA1c >6. 5%, n=12), 12 mice in each group. Using IE mice as fecal donors and SE mice as recipients, fecal microbiota transplantation was performed between the two groups, the FMT group (given fecal bacterial suspension, n=5) and the Sham group (given equal amounts of sterile saline, n=5). The intestinal microorganisms of mice in the IE group (donor) and SE group (recipient) were also analyzed for differences. To assess the protective effect of FMT on drug efficacy and T2DM, and to explore the underlying mechanisms.

**Results:**

After 10 weeks, compared with the control group, the HbA1c of the experimental group was significantly reduced, still, the level of HBA1c of the mice in the unsatisfactory group was significantly higher than that in the ideal group. Compared with the unsatisfactory group, fasting blood glucose, 2h postprandial blood glucose, blood glucose AUC and body weight were significantly reduced in the ideal group. 16srDNA sequencing showed that the levels of Bacteroidota, Akkermansia, Parabacteroides, Bifidobacteria and other bacteria in the ideal efficacy group were significantly higher than those in the non-ideal efficacy group (P<0.05). The levels of Firmicutes, Romboutsia, Clostridium, Turicibacter and other bacteria in the unsatisfactory group were significantly higher than those in the ideal group (P<0.05). The dominant flora of mice in the ideal drug efficacy group was negatively correlated with HbA1c and blood sugar, and the dominant flora of mice in the unsatisfactory drug efficacy group was positively correlated with pro-inflammatory factors such as blood sugar. Moreover, FMT treatment significantly improved the efficacy of PEX168 and liver steatosis in the group with unsatisfactory efficacy.

**Conclusion:**

In summary, we used the combined method of 16S rDNA and metabolomics to systematically elucidate the efficacy of microflora on PEX168 and the possible mechanism of FMT in treating T2DM by PEX168. The difference in intestinal flora between individuals can affect the therapeutic effect of drugs. Moreover, FMT therapy can affect multiple metabolic pathways and colonization of beneficial bacteria to maintain the drug’s therapeutic effect on T2DM mice.

## Introduction

Globally, diabetes is becoming increasingly prevalent, according to the International Diabetes Federation (IDF). It is predicted that 642 million people will have diabetes by 2040 (1, 2), with T2DM comprising over 90% of these cases. T2DM is a chronic metabolic condition primarily characterized by insulin resistance (IR) and a reduction in insulin production. Once without effective treatment, it might lead to a composite of microvascular or macrovascular complications, for instance chronic kidney disease, diabetic eye disease, and cardiovascular disease (CVD); these complications often impose a significant financial and medical burden on healthcare systems globally (1, 3).

T2DM is currently treated primarily with oral hypoglycemic drugs and insulin. While thypoglycemic traditional medicines, including metformin, sulfonylureas, thiazolidinediones, α-glucosidase inhibitors, and insulin, may exert hypoglycemic effects through different mechanisms, they are prone to adverse impacts, including hypoglycemia, weight gain, severe ketonuria, and lactic acidemia (4, 5). Therefore, clinically, there is an urgent need for drugs with stable glucose-lowering effects and a low incidence of adverse effects. Drugs that target the GLP-1 receptor (GLP-1R), such as liraglutide and losenatide, have garnered significant attention due to their ability to effectively promote insulin secretion and enhancing glucose homeostasis (6, 7); these agents offer promising options for alleviating and treating diabetes mellitus and its associated complications.

Glucagon-like peptide-1 (GLP-1) is the most potent intestinal peptide for insulin secretion identified so far, and it is secreted primarily by L cells in the ileum and colon, encoded by the human glucagon gene (8). Since 1985, studies have demonstrated that GLP-1 functions as an insulinotropic agent, reducing blood glucose levels (9). Moreover, GLP-1 agonists have also been shown to suppress appetite and delay gastric emptying, thereby contributing to weight control (10). Furthermore, GLP-1 agonists have demonstrated a lower risk of hypoglycemic events compared to insulin, and some have even exhibited cardiovascular protective effects (11), As a result, GLP-1 agonists have emerged as a promising new class of drugs with significant growth potential in the treatment of diabetes. However, naturally occurring GLP-1 is hydrolyzed by dipeptidyl peptidase 4 (DPP4), resulting in a half-life of less than 5 minutes, which limits its clinical effectiveness (12). and the convenience offered by long-acting weekly preparations has dramatically simplified and enhanced long-term glycemic control for diabetes patients (11, 13, 14). PEX168 was the only GLP-1RA to enhance the therapeutic dose without increasing the risk of hypoglycemia. As a result, drugs like PEX168 have emerged as up-and-coming hypoglycemic agents.

In healthy individuals, A stable gut microbiota composition is paramount in safeguarding the gut barrier’s integrity and maintaining inflammatory balance in healthy individuals. The gut stands out as a pivotal organ responsible for endogenous GLP-1 secretion. Which also serves as a prime target for the effects of exogenous GLP-1 receptor agonists (15). It plays an important in the regulating of glucose homeostasis in the body. A diverse array of microorganisms populates the guts of all postnatal animals, humans included. The human gut, harbors approximately 1014 microorganisms belonging to 500 distinct species, all residing on the intestinal epithelial barrier (16). These gut microorganisms are important and engage in diverse physiological and metabolic functions within the human body. They play a crucial role in facilitating intestinal digestion and absorption of nutrients, regulating the expression of genes about development, differentiation, angiogenesis, and energy metabolism through numerous pathways (17), and function as environmental modulators, regulating lipid metabolism and influencing the epigenetic inheritance of the host (18).

Furthermore, recent studies have revealed that specific host proteins play a pivotal role in shaping, The composition of the intestinal flora (16). Evidently, the intestinal environment is a product of the intricate interaction between the host and its intestinal microflora. Any disruption in the balance of the intestinal flora can lead to gastrointestinal diseases like diarrhea, constipation, and chronic enteritis. Additionally, such dysbiosis has been linked to a range of other health issues, including obesity, ageing, metabolic syndrome, cardiovascular disease, and diabetes (19). A 2017 study published in (Cell Metabolism) revealed that a particular class of bacteria in the ileum can influence the glucose-lowering effects of GLP-1 (20). Some patients, however, demonstrate resistance to GLP-1 drugs, a condition known as GLP-1 resistance (14, 15, 21). The underlying mechanisms of this resistance remain incompletely understood and require further exploration. Given mild gastrointestinal adverse reactions associated with PEX168 PEX168 usage, specific subgroups of the intestinal flora might contribute to the drug’s efficacy. Additionally, it has been observed that patients undergoing PEX168 treatment may experience changes in their intestinal flora, Which can manifest as symptoms such as diarrhea and vomiting. This suggests that the effectiveness of PEX168 could be influenced by individual variations in the patient’s intestinal flora, pointing to a possible interaction between the drug and the intestinal flora. Furthermore, PEX168 may also induce modifications in specific subspecies of the intestinal flora.

We observe that such suboptimal GLP-1 pharmacodynamics is not exclusive to animals treated with GLP-1 receptor agonist (GLP-1RA) drugs, it can also occur with DPP4 inhibitor drugs (22). It is suggested that diabetes drugs designed based on the GLP-1 signaling pathway exhibit a certain degree of individual variability in their pharmacodynamic effects. Therefore, our research team aims to delve into the reasons for individual differences in drug efficacy by focusing on type 2 diabetic mice that respond poorly to treatment. We hypothesize that manipulating the intestinal flora through fecal microbiota transplantation could enhance the glucose-lowering effect of PEX168. Based on our hypothesis, the individual differences in gut microbiota play a crucial role in determining the efficacy of PEX168 in treating T2DM. We postulate that FMT could potentially enhance the drug’s effectiveness in suboptimal individuals by modifying and reconstituting their intestinal microbiota. To validate this, we have established a mouse model of T2DM to identify mice that exhibit both ideal and suboptimal responses to PEX168. To assess whether FMT can index improve the drug’s efficacy in the suboptimal responders. And elucidate the underlying mechanism involved. and further explore the potential role of the microbiotic-intestinal axis in the pathogenesis of T2DM and the mechanisms underlying drug efficacy.

## Materials and methods

### Materials

PEX-168 was supplied by Haosen Pharmaceutical Co. Ltd.(Jiangsu, China), Batch No.: H20190025. The specification is 0.5 ml (0.2 mg)/bottle, stored at 4~8°C. Streptozotocin (STZ): sigma product (S-0130) is stored at -20 °C and before use, it is configured into a concentration of 1% STZ solution with a citrate buffer ice bath of 0.1 mol/l (PH=4.3). The high-fat diet was purchased from Synergy Pharmaceuticals Bioengineering Co. Ltd (Jiangsu, China). Elisa and GHbA1c kits were purchased from Beinlai Biotech Co. and the blood glucose meter is from Roche in Germany.

### Animal and experimental design

50 C57BL/6 male mice (4 weeks old) were purchased from the Laboratory Animal Center of Shanxi Medical University (Taiyuan, Shanxi Province, China). The mice were bred in a 12-h dark-night cycle SPF room in standard cages (5 mice/cage) at a temperature of 22 ± 1°C. All animals had ad libitum access to water and standard chow. All animals experiments were approved by the Animal Care and Use Committee of Shanxi Medical University (Approval No. DW2023020).

**Figure 1:**
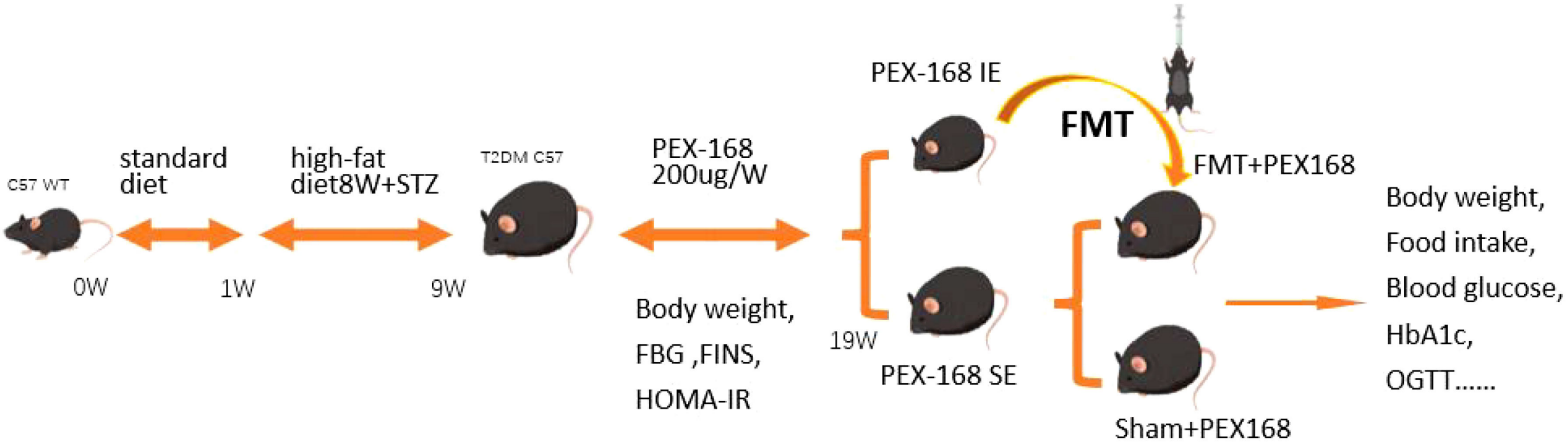
The design of the animal experiments.

The General flow chart of animal experiments is shown in **Figure 1**. After 1 week of adaptive feeding, the mice were fed a high-fat diet (58% fat, 5.6% carbohydrates, and 16.4% proteins) and continued to feed for 8 weeks. The mice were deprived of water and injected the next day with STZ at a dose of 60 mg/kg. Random blood glucose was measured after 7 days, and mice with values ≥16.7 mmol/l indicated successful T2DM modelling, and there were 40 mice in total. All mice were randomly divided into two groups: (the T2DM group, n=10) and (the PEX168 treatment group, n=30). Mice in the experimental group were subcutaneously injected with PEX168 (200ug/kg) once a week, and mice in the control group were subcutaneously injected with an equal volume of saline for 10 weeks. These mice were monitored weekly for indicators of glucose metabolism. After 10 weeks, the mice in the drug intervention group were divided into the IE group (HbA1c ≤ 6. 5%) and the SE group (HbA1c>6. 5%). Excluding the dead mice in the experiment, 12 remaining mice were divided into IE group and SE group according to HbA1c value. Next, We randomly divided the SE group into the FMT group(n=5) and the Sham group(n=5). FMT group mice were gavaged with 200ul of fecal bacteria solution per day, while the sham group was given sterile normal saline simultaneously. Both groups’ mice were injected with PEX168 200ug/kg subcutaneously weekly for 8 weeks. The mice were given weekly measurements of glucose metabolism, such as blood glucose and body weight.

### Fecal microbiota transplantation

Mice in the IE group were used as FMT donors. Collect fresh fecal pellets in the morning and freeze them immediately in liquid nitrogen. The frozen fecal samples were thawed in a constant temperature water bath at 37.5°C for 10 minutes, and then the fecal samples were suspended in sterile normal saline with a dilution ratio of 200mg feces to 2ml volume, and mixed until there were no significant large feces particles. Subsequently, the large particles in the stool are removed with a sterile 200-mesh screen, and the filtrate is then passed through a 400-mesh and 800-mesh screen to remove undigested food and more minor particulate matter. Centrifuge the suspension at 1000×g for 3 minutes to remove the insoluble material, and centrifuge the dissolved feces at 1000 g(4°C) for 3 minutes to separate the bacterial components from the residual solids. Finally, the centrifuged feces suspension is packed on a clean workbench and stored at -80°C (23) degrees for future use.

### Indicators and measurements

General condition: Mice were observed every week, and their appearance, urine, feces, activity, gait, spirit, appetite, and any abnormalities were monitored.

Measurement of body weight, food intake, and blood glucose: Weight changes in mice were measured weekly. Measure the amount of food provided and record the weight of the remaining feed the next day, excluding any debris. This information is used to calculate daily food intake. The mice were not allowed to drink any water and fasted for 12 hours. The following morning, FBG levels were measured with a tail cutter and a hand-held glucose meter once a week.

Serum Biochemical Analysis: Blood was collected through the orbital vein of all mice, centrifuged at 4°C (3,000× g) for 15 min, and the supernatant was preserved. The fasting insulin (FINS) and HbA1c level were measured by mouse ELISA kit. Insulin resistance index Homa-IR=FINS x FBG/22.5 was calculated. after fasting for 12 hours. Mice were given 2 g/kg of glucose via gavage. The blood glucose levels of mice in each group were measured at 0, 0.5, 1, and 2 hours, respectively, and the changes in the oral glucose tolerance test (OGTT) were compared.

Histopathological analysis: Liver and pancreas tissues were fixed in a 4% paraformaldehyde solution and dehydrated with araffin-embedded. The embedded liver sections were stained with hematoxylin and eosin (H&E) and Oil Red O (ORO) (24). The pancreatic tissues were stained with H&E, and the pancreatic islets of GLP-1R were analyzed by immunohistochemical. Liver and pancreatic tissue images (200×) were obtained under an inverted microscope.

### DNA extraction and 16S rDNA gene sequencing

The feces were collected from mice in the pharmacologically ideal and less suboptimal groups for microbiota sequencing analysis. After placing each mouse in a separate empty autoclave cage, we collected 6 to 8 fresh fecal pellets per mouse which we immediately put into sterile EP tubes. All fecal samples were frozen and stored at -80°C until further analysis (25). Following the manufacturer’s instructions, We extracted microbial genomic DNA from these samples using the QIAamp Fast DNA Stool Mini Kit (Qiagen, Germany). To amplify the V3-V4 region of microbial 16S DNA we utilized paired primers consisting of a forward primer (5 ‘ -CCTACGGGRS gcagcag3 ‘) and a reverse primer (5 ‘ - GGACTACVVGGG TATCTAATC-3 ‘). The following conditions were used:95°C for 3 minutes, followed by 30 cycles at 98°C for 20 seconds, then 58°C for 15 seconds, at 72°C for 20 seconds, and finally an extension at 72°C for 5 minutes. All quantitative amplicons were pooled at uniform concentrations for Illumina MiSeq sequencing (Illumina, Inc., CA, USA). The experiments, including DNA extraction, quality assessment, library construction, and high-throughput sequencing, were performed by Jiangsu-Suichuan Biotech (Jiangsu-Suichuan, China).

Alpha diversity indices (Chao 1 and Simpson indices) were calculated using QIIME 2 (V1.9.1) (26), and differences between the 3 groups were analyzed using the Kruskal-Wallis test in R (V3.5.1). Diversity analysis was performed using weighted UniFrac distances. Subsequently, differences between groups were compared using Adonis and displayed using principal coordinate analysis (PCoA). In addition, the analysis of similarity (ANOSIM) method was used to compare differences between groups. Linear discriminant analysis (LDA) was used to compare differences in microbial abundance at the level of different taxonomic units using LDA EffectSize Tools (V1.0).

### Statistical analysis

Data in this study were analyzed using R software (version 4.1.0) and SPSS software (version 26.0). And GraphPad Prism software (version 8.0.2). All data were expressed as mean ± standard deviation (SD). One-way ANOVA was used for data conforming to a normal distribution, and a t-test was used to compare the two groups. The non-parametric test (Kruskal Wallis) was used to analyze the data that does not meet the normal distribution. ns *P* > 0.05 (not significant); * 0.01 < *P* < 0.05; ** 0.001 < *P* < 0.01; *** *P* < 0.001.

## Results

### Changes in glucose metabolism levels in mice of IE and SE groups

**Figure 2:**
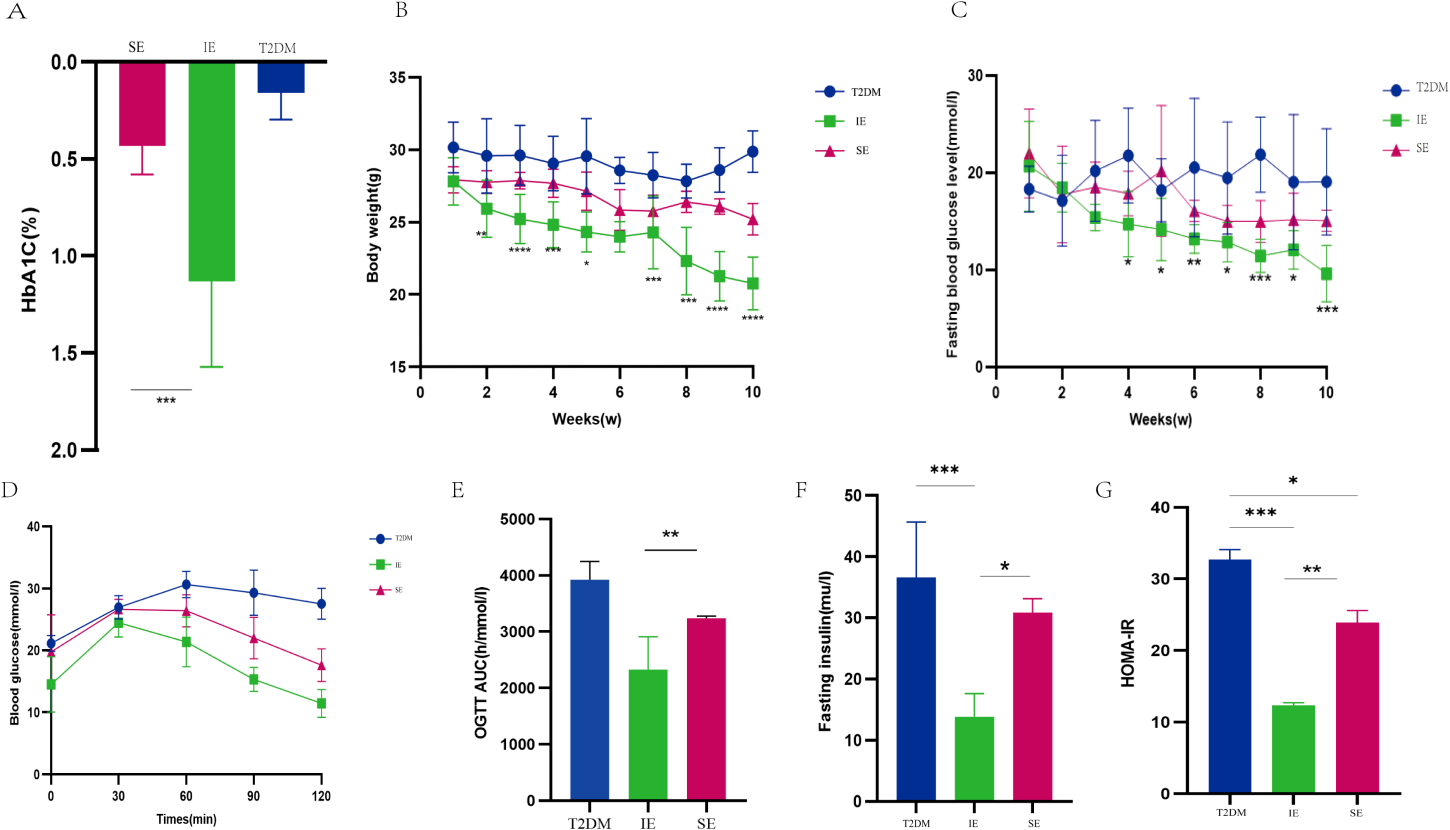
Screening and inclusion of mice with ideal and unsatisfactory efficacy of PEX168. **(A)**Baseline HbA1C level; **(B)** fasting blood glucose (FBG); **(C)** body weight (g); **(D, E)** oral glucose tolerance test (OGTT) and AUC at 12th week; **(F)** fasting insulin level; **(G)** HOMA-IR. Data are expressed as mean ± SD. Data were expressed as mean ± standard deviation (means ± s.d.) using average values. **(G)** Insulin resistance index. (^*^ *P* < 0.05, ^**^ *P* < 0.01, ^***^ *P* < 0.001). **** refers to data with P<0.001 in the statistical analysis.

There were no significant differences in preexperimental HbA1c levels among the three groups of mice, **Figure 2A** shows the percentage reduction in HbA1c relative to baseline in three different groups of mice after drug intervention, HbA1c was 1.25% lower than baseline in the IE group, 0.15% lower in the T2DM group, and 0.45% lower in the SE group. Compared with the SE group, HbA1c in the IE group was significantly decreased, and the difference between the two groups was statistically significant (*P* < 0.001). Both IE and SE groups had lower body weight and blood glucose than the T2DM group. Compared with the SE group, body weight and FBG in the IE group decreased significantly, and there was a difference between the two groups (**Figures 2B, C**, * 0.01 < *P* < 0.05; ** 0.001 < *P* < 0.01; *** *P* < 0.001). As shown in **Figures 2D, E**, both the T2DM and SE group mice showed severe glucose intolerance, In contrast, the glucose tolerance of mice in the IE group was significantly improved, indicating that the ability of mice in the IE group to regulate glucose damage was enhanced considerably. Serum biochemical tests showed that FINS (*P* < 0.05) and HOMA-IR (*P* < 0.001) of mice in the IE group were significantly higher than those in T2DM and SE groups, as shown in **Figures 2F, G**.

**Figure 3:**
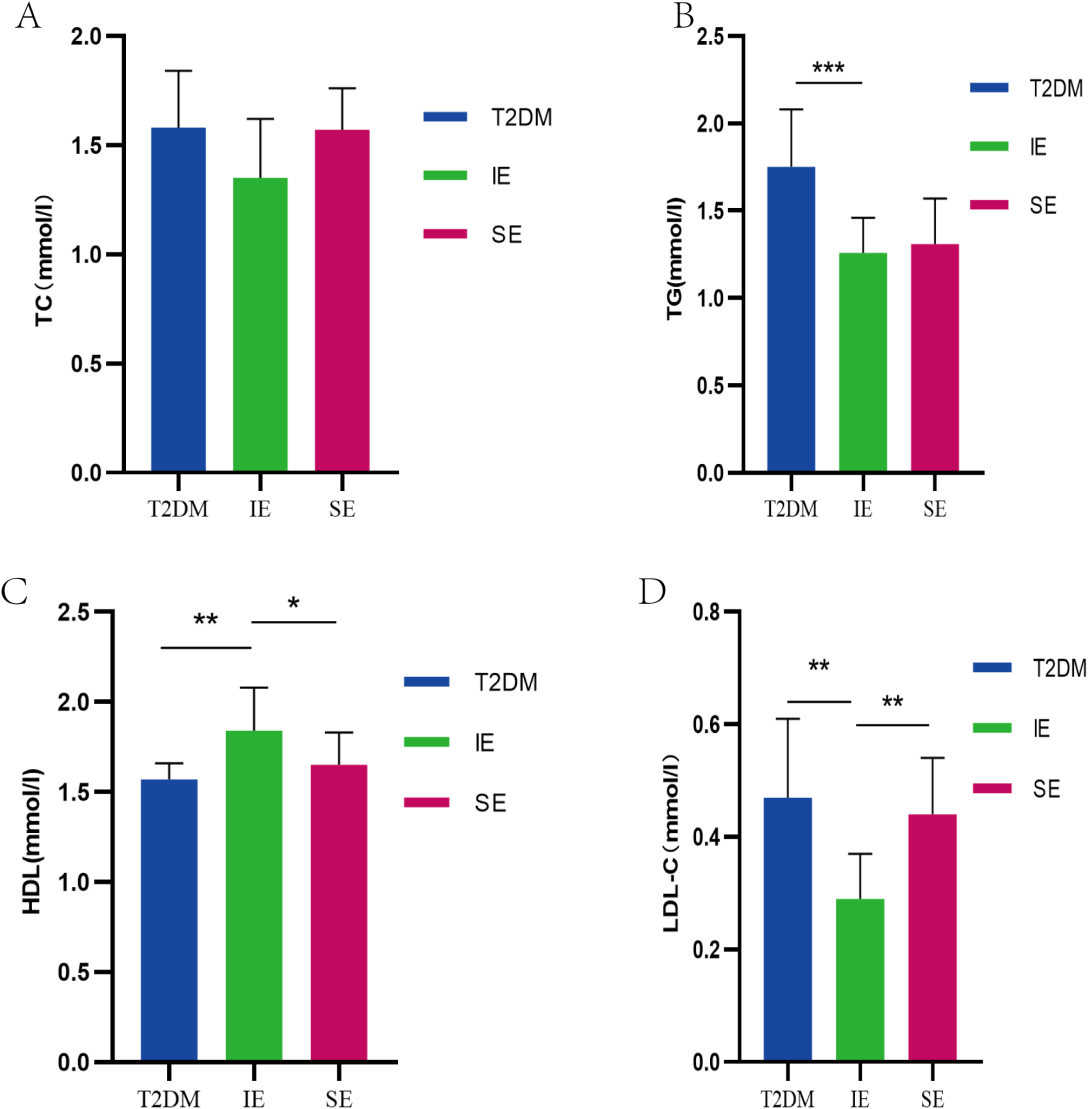
**(A)** TC level of mice in each group during the experiment; **(B)** TG levels of mice in each group during the experiment; **(C)** The level of HDL-C in each group of mice during the experiment; **(D)** LDL-C level of mice in each group during the experiment (*P<0.05; **P<0.01; ***P<0.001; ***P<0.0001).

Long-term high-fat diet will lead to an increase in TC, total TG and LDL-C content in the body. The effect of PEX168 on serum lipids is shown in the figure. Serum TC and TG levels in T2DM mice were significantly increased; however, after PEX168 treatment, the changes of serum TG were significantly reversed (p < 0.01, **Figures 3A, B**), especially in the IE group, which significantly increased serum HDL-C level (**Figure 3C**) and decreased LDL-C level (**Figure 3D**). Effective regulation of glucose and lipid metabolism disorders in T2DM mice. (*P<0.05; **P<0.01; ***P<0.001; ***P<0.0001).

### Intestinal flora analysis among each group

**Figure 4:**
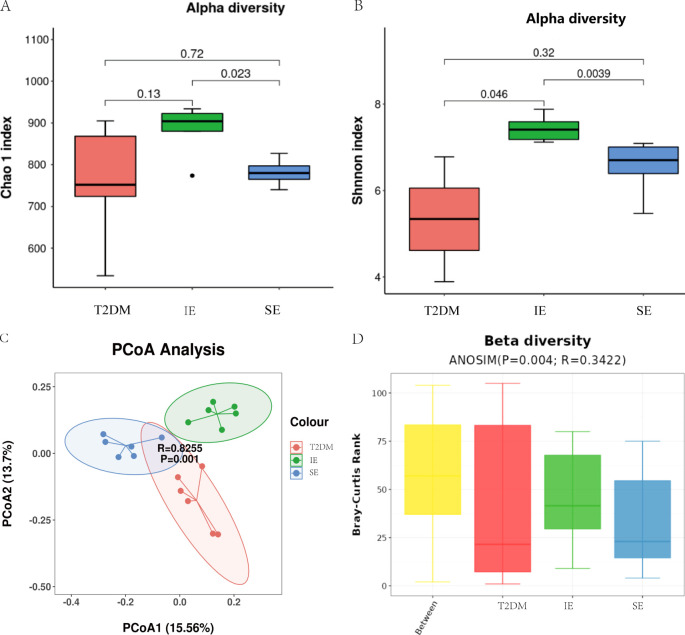
Diversity analysis of intestinal flora **(A)** Analysis of alpha diversity of gut microbiota by Chao 1 analysis. **(B)** Analysis of alpha diversity of gut microbiota by Shannon analysis. **(C)** PCoA plots of beta diversity based on weighted UniFrac analysis in different groups. **(D)** Beta diversity based on weighted UniFrac ANOSIM analysis in different groups.

The fecal microbiota of three groups of mice was analyzed by 16S rDNA gene sequencing. First, an alpha-diversity analysis was performed to assess the richness and diversity of bacterial species, as shown in **Figures 4A, B**. The Chao 1 index (*P* = 0.18) and Shannon index (*P* = 0.011) in the IE group were higher than those in the T2DM and SE group. In addition, alpha diversity was significantly different between the IE and SE groups (Chao 1 index: *P* = 0.023; Shannon index: *P* = 0.0039; **Figures 4A, B**). As shown in **Figure 4C**, the microbial community of the T2DM group was significantly different from that of the IE and SE groups. Principal component analysis revealed that the microflora of IE group and the SE group had apparent clustering, and the microflora of the T2DM group had significantly more flora than other groups. It is suggested that PEX168 may affect the intestinal flora structure of mice. In addition, a measure of beta diversity on weighted UniFrac distances indicated similarity in microbiota composition between groups. Adonis test and ANOISM analysis of variance (R = 0.3422, *P* = 0.004; **Figure 4D**) showed that there were significant differences in the bacterial flora between the three groups.

**Figure 5:**
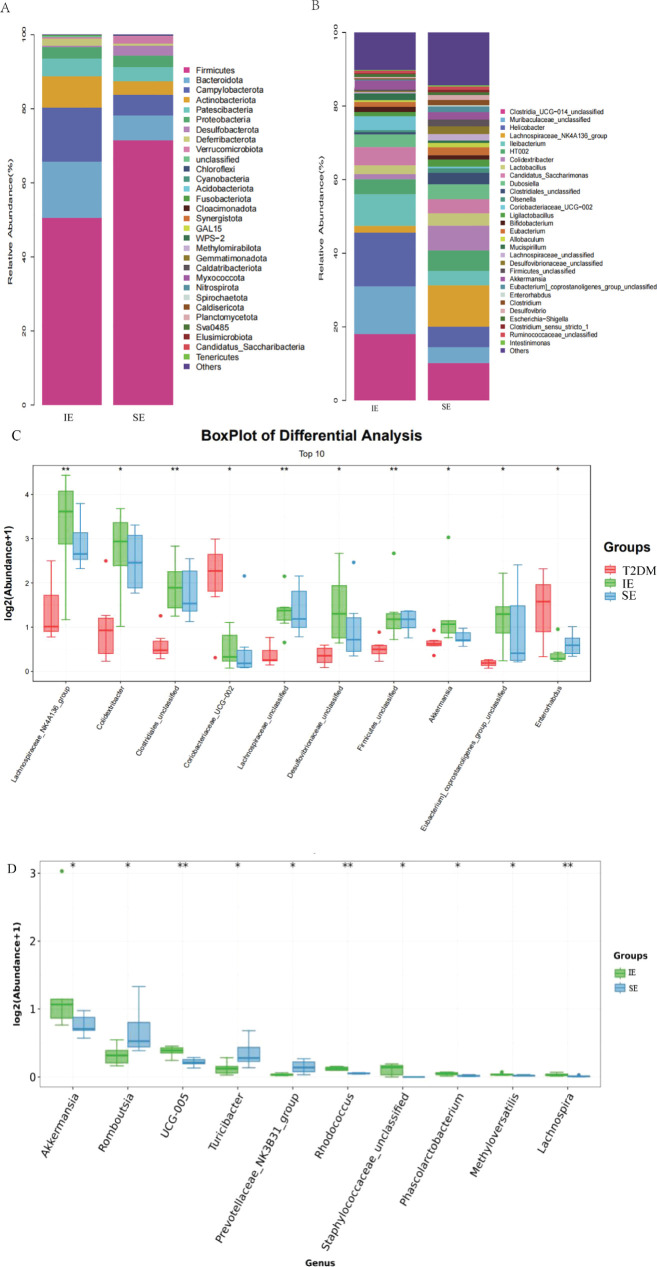
**(A)** The relative abundance of the gut bacterial phylum in each group. **(B)** The relative abundance of the gut bacterial genus in each group. **(C, D)** (I) Relative abundances of significantly altered bacterial genera by boxplots. Comparisons between groups were made using the Wilcoxon rank sum test (^*^ *P* < 0.05, ^**^ *P* < 0.01).

The microbial composition at the phylum and genus levels is shown in **Figures 5A, B**, respectively, in which a total of 30 kinds of bacterial were detected, and Firmicutes(mean value 62.87%) and Bacteroides(mean value 11.51%) were dominant in the three groups (**Figure 5A**). At the phylum level, the abundance of Firmicutes in the IE group was significantly lower than that in the SE group. The abundance of Patescibacteria and Proteobacteria was similar in the three groups. Compared with SE group, the abundance of Firmicutes and Bacteroidota, Campylobacterota, Desulfobacterota and Verrucomicrobiota in group IE they were increased, while the abundance of Firmicutes and Deferribacterota decreased. The F/B ratios of the T2DM group, IE, and SE groups were 10.62, 3.31, and 5.24, respectively. The F/B ratios of the IE and SE groups were much lower than that of the T2DM group, and the F/B ratio of the T2DM group was about twice that of the IE group. In addition, among the 30 genera with the highest abundance, Clostridia_UCG-014, Helicobacter were enriched in the T2DM group, Meanwhile, Akkermansia, Lachnospiraceae_NK4A136 and Colidextribacter were significantly enriched in the IE group. Allobaculum and Olsenella were significantly enriched in the SE group (**Figure 5B**). We further select the top 10 genera and display their details using box plots. Compared to the experiment group, the abundance of Coriobacteriacae and Enterorhabdus in the T2DM group increased, while the abundance of most other bacterial genera decreased. The comparison of bacteria at different taxa levels showed that the overall differences of most bacterial genera among the three groups were statistically significant (^*^ *P* < 0.05, ^**^ *P* < 0.01; **Figure 5C**). Compared with the SE group, the abundance of Akkermansia, UCG_005, Rhodococcus, Staphylococcaceae, and Phascolarctobacterium showed significantly higher in IE group had increased considerably. The abundance of Romboutsia, Turicibacter, Prevotellaceae, and Lachnospira was significantly decreased (^*^ *P* < 0.05, ^**^ *P* < 0.01; **Figure 5D**).

**Figure 6:**
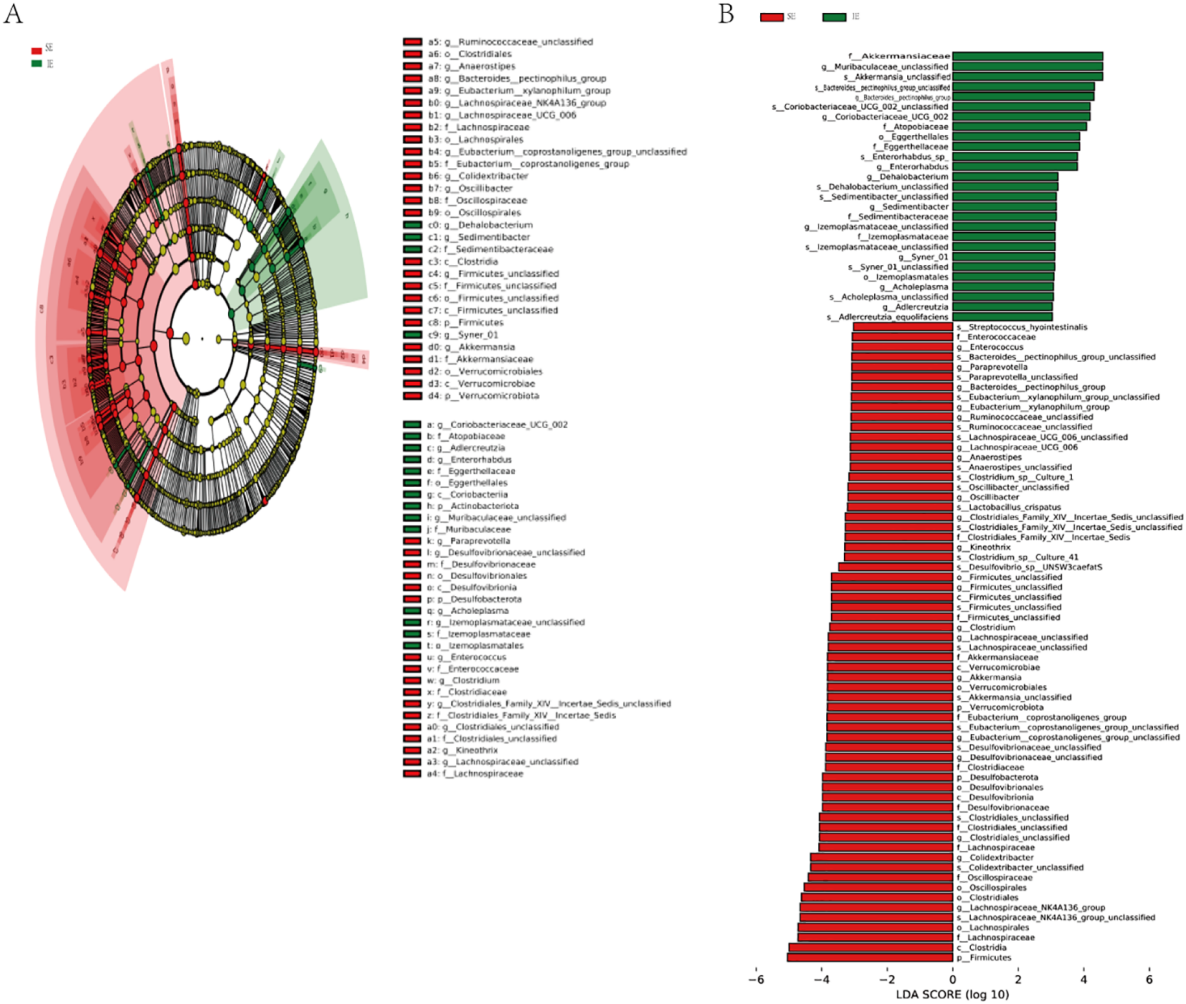
LEfSe discriminant analysis of gut microbiota in 3 groups. **(A)** Comparison of taxonomic abundances using LEfSe. The circles radiating from inside to outside represent the taxonomic levels from phyla to species. The dots located on individual circles represent different classification levels of bacteria. The size of each dot is proportional to its taxonomic abundance. The dot colors match with those of 3 experimental groups. **(B)** Histogram of linear discriminant analysis (LDA) represented significant difference in abundance of gut bacteria between each groups. A high LDA score indicates great effect of species abundance on the difference between groups.

To further identify the specific bacteria in different groups, the microbiota composition of each group was compared using linear discriminant analysis effect size (LEfSe). The results revealed the presence of a total of 48 taxa across the three groups (**Figure 6A**). There were distinct differences in the composition of the gut flora among the groups. Furthermore, the major taxa exhibiting significant differences among the three groups were identified by LEfSe (*P* < 0.05; LDA > 3.0). The top 3 taxa enriched in the fecal flora of the T2DM group were f_Lachnospiraceae, f_Clostridiaceae, and g_Lachnospiraceae (**Figure 6B**). Additionally, the bacterial taxa of the IE and SE groups were compared by the same method. The abundance of Akkermansiaceae and Verrucomicrobiota was higher in the IE group. While Peptostreptococcaceae, Romboutsia, and Turicibacter were more abundant in the SE group.

### FMT treatment improvement drug treatment effect in SE group mice

**Figure 7:**
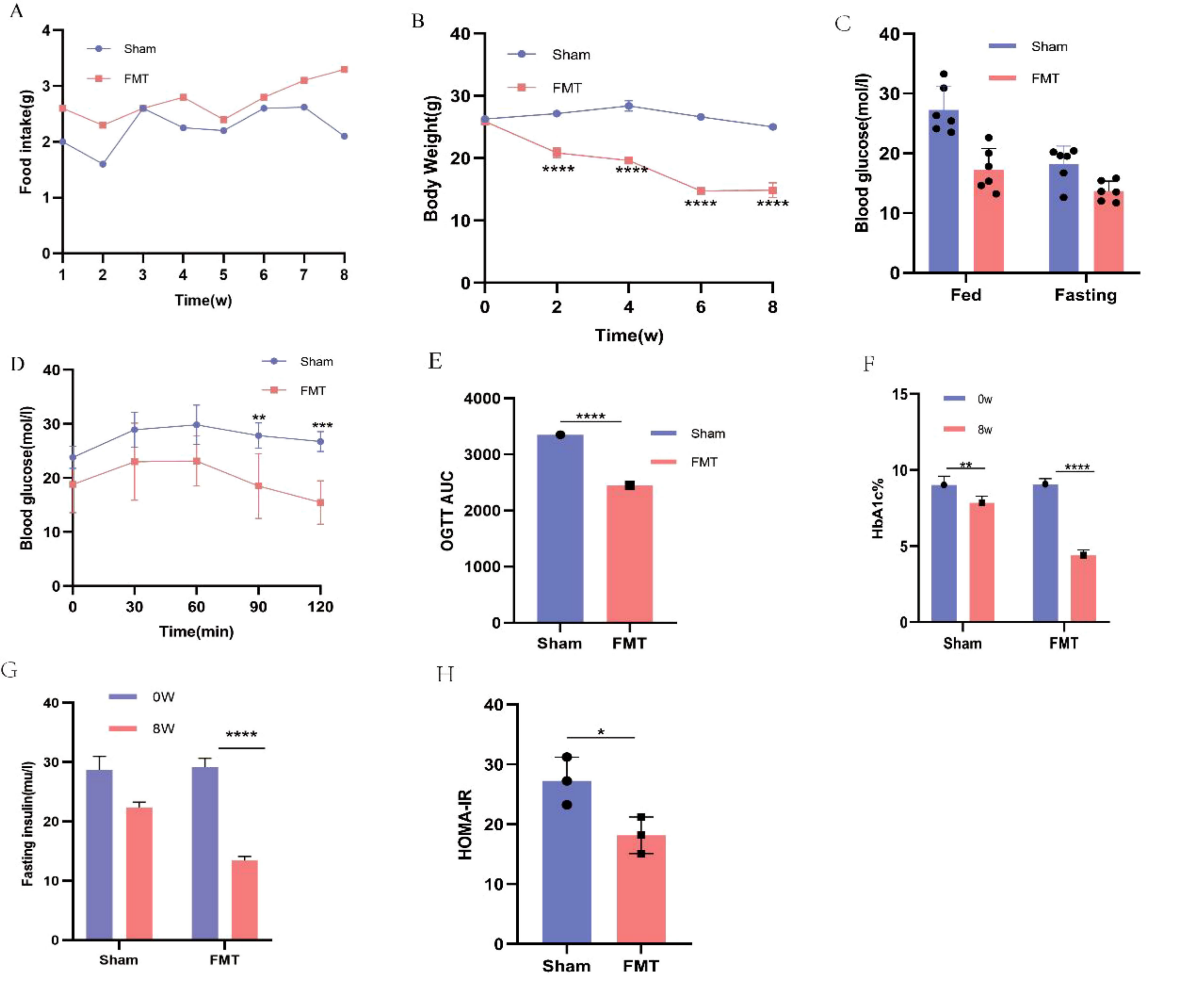
Comparison of food intake, body weight, blood glucose and serum biochemical glucose metabolism indexes between FMT group and Sham group. **(A)** body weight; **(B)** food intake; **(C)** fasting blood glucose (FBG) and Randome Blood glucose; **(D, E)** oral glucose tolerance test (OGTT) and AUC ;**(F)** HbA1C (%); **(G)** fasting insulin; **(H)** HOMA-IR;. (^*^ *P* < 0.05, ^**^ *P* < 0.01, ^***^ *P* < 0.001). **** refers to data with P<0.001 in the statistical analysis.

There was no significant difference in baseline values between the two groups. HbA1c, FBG, OGTT, body weight and other vital indicators were carefully observed and recorded before and after transplantation. As shown in the figure, compared with the Sham group, the FMT group had significantly lower food intake (**Figure 7A**), body weight (**Figure 7B**), random glucose and FBG (**Figure 7C**). Additionally, the sham group exhibited severe glucose intolerance on the OGTT (**Figures 7D, E**). While FMT treatment significantly improved glucose tolerance in T2DM mice. Furthermore, HbA1C levels were reduced considerably in the FMT group compared to the sham group, with a decrease of approximately 1.4% (**Figure 7F**). Moreover, FMT treatment effectively reduced FINS levels in T2DM mice (P < 0.05; **Figure 7G**). Similarly, HOMA-IR was significantly reduced in the FMT group (P < 0.001; **Figure 7H**). In summary, these findings indicate that FMT treatment improves the drug efficacy of PEX168, resulting in improved glucose tolerance, reduced HbA1c levels and insulin resistance, and a protective effect against T2DM.

**Figure 8:**
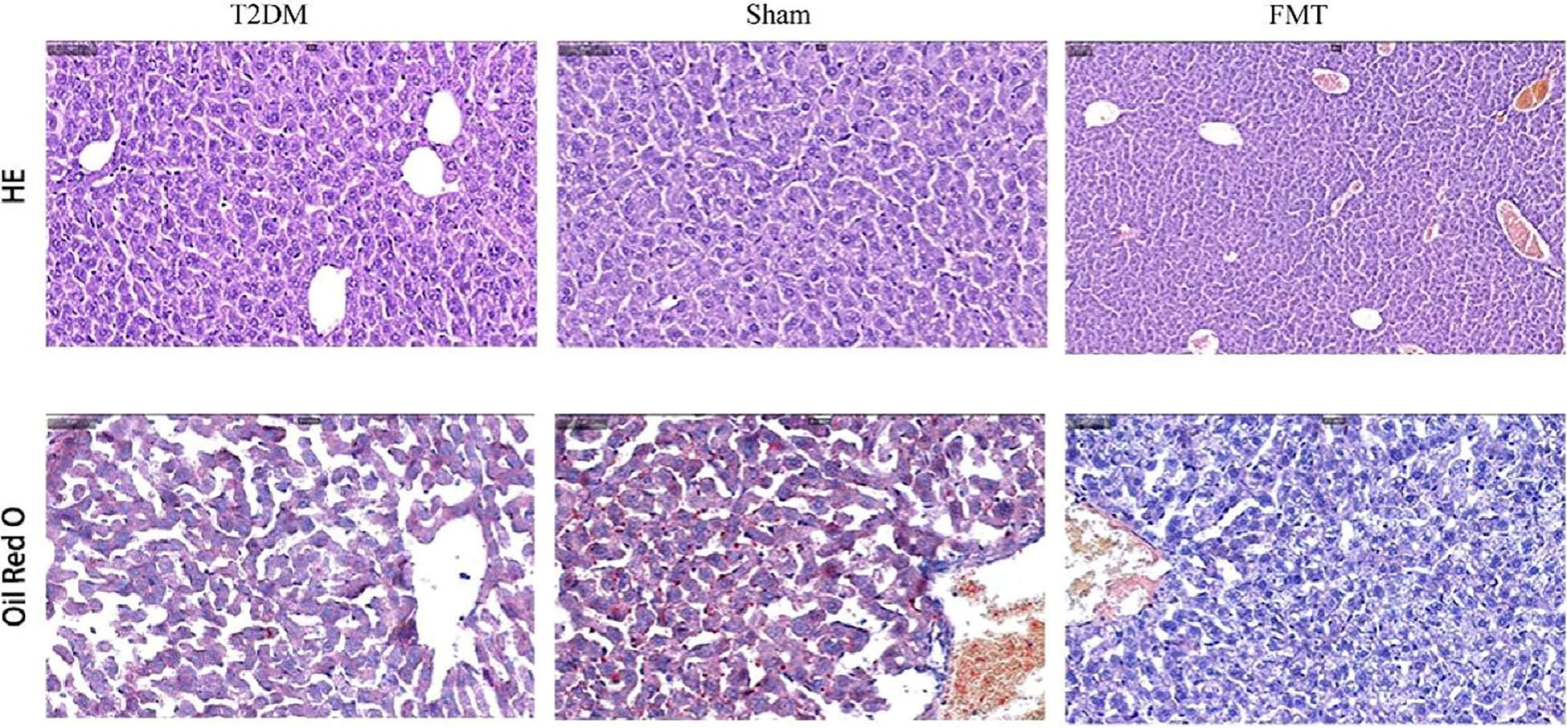
Representative images (200×) of liver tissue H&E, ORO.

As shown in **Figure 8**, T2DM mice exhibited cytoplasmic vacuolation and hepatocyte necrosis. In addition, ORO staining is a standard experimental method to observe and analyze lipid accumulation in the liver. Oil red dye can directly react with Triglyceride (TG) in the liver to make it red, which can more intuitively observe lipid accumulation in the liver of mice. In the T2DM group, the red area was more significant, and the ORO-positive area was significantly increased, indicating that there was a large amount of lipids in the liver of mice, and steatosis was more serious. Compared with the Sham group, the vacuolar degeneration and liver injury of mice in the FMT group were significantly reduced, suggesting that FMT treatment improved liver lipid accumulation in the FMT group.

**Figure 9:**
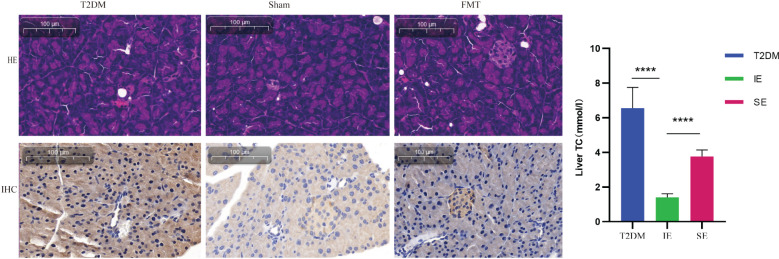
Representative images of H&E in pancreatic tissue (200×); Representative image of pancreatic islet with GLP-1R immunohistochemical staining. **** refers to data with P<0.001 in the statistical analysis.

As shown in **Figure 9**, as observed in this study, T2DM mice had swollen islets with irregular morphology and blurring. The morphologies of the ialets of mice in the FMT group showed improvement compared to the Sham group. PEX168 is an agonist of GLP-1R, and FMT treatment can improve the drug efficacy of PEX168 and promote the synthesis and release of insulin by binding to GLP-1R. Immunohistochemical pancreatic pancreatic GLP-1R showed that PEX168 significantly increased pancreatic GLP-1R levels compared to T2DM mice and promoted insulin secretion. In addition, FMT significantly increased pancreatic GLP-1R levels in mice compared to the Sham group, can repair damaged islet cells and promote insulin secretion from pancreatic β-cells, thus exerting hypoglycemic effects.

## Discussion

Many studies imply that gut microbiota dysbiosis is linked with T2DM development (17, 27). The gut microbiota is also crucial in regulating drug efficacy (28, 29). However, there are still gaps in research on the potential interactions between the human gut microbiota and GLP-1 and their therapeutic responses in patients with T2DM. To address this, we used an STZ-induced T2DM mouse model to investigate the effect of individual differentiated gut microbiota on the efficacy of the T2DM drug PEX168. We further evaluated the protective effect of FMT treatment on T2DM to explore possible mechanisms. Our funding indicates that FMT treatment may have altered the structure of the intestinal flora in mice, which in turn affected the drug efficacy of PEX168 and improved glycolipid metabolism in mice. A specific group of gut microbiota was found to influence GLP-1 resistance (20), and many hypoglycemic drugs result in changes of intestinal microbiota. Our current study showed that PEX168 had different therapeutic effects in other individuals. Compared with the SE group, the drug intervention significantly alleviated the higher FBG levels, reduced body weight, and improved glucose tolerance and insulin resistance in the IE group. The SE group partially alleviated these symptoms and could potentially mitigate HbA1C in mice, but it could not achieve the ideal goal of drug therapy, and the impact was far less than that of the IE group mice. In addition, FMT treatment can enhance the effect of PEX168 on blood glucose and improve the glucose and lipid metabolism of SE group mice. In summary, there may be interactions between intestinal microbiota and drugs, and FMT treatment may alter the microbiota ecology, thereby improving the efficacy of drug therapy.

GLP-1RAs are novel hypoglycemic agents that have emerged in recent years and are now the focus of clinical studies for treating T2DM because they have specific outstanding advantages (30, 31). GLP-1RA can enhance glucose-dependent insulin secretion, inhibit glucagon secretion, slow stomach peristalsis, and reduce body weight by increasing satiation and decreasing appetite (32, 33). It has become a clinical research hotspot in the treatment of T2DM. Importantly, GLP-1RA have a lower risk of hypoglycemia with treatment or without sulfonylureas or insulin (34, 35). PEX-168 is the first self-development long-acting GLP-1RA in China, and It is widely used because of its advantages in reducing blood glucose levels and weight loss in patients. However, in clinical application, it has been found that not all patients achieve the desired treatment goals (25, 36). A study on reasons for GLP-1RA found that 25.0% of those who discontinued GLP-1RA reported “no improvement in quantity” as the primary reason for discontinuation, i.e., lack of glycemic improvement, lack of weight loss, etc. (36). This is consistent with our findings. Compared with mice in the IE group, the SE group failed to achieve the desired therapeutic goals of lowering blood sugar and losing weight. The biological response to GLP-1RA treatment may vary significantly depending on the individual’s unique physiology characteristics.

We know that oral hypoglycemic drugs, especially those that target the intestinal system, reach the gastrointestinal tract and meet the gut microbiota. Changes in gut microbiota or functional capacity will often be observed in individuals with T2DM. For example, acarbose treatment significantly increased the abundance of intestinal bifidobacteria in T2DM patients (37). In turn, gut flora plays a vital role in regulating drug efficacy. The interaction between intestinal flora and hypoglycemic drugs is complex and bidirectional. A recent study found that GLP-1 levels can be elevated by changing the environment (38). Grasset et al. demonstrated that the gut microbial ecosystem is critical to maintaining GLP-1 sensitivity, and they found that high-carbohydrate-high-fat obese diabetes (HC-HFD) mice were severely resistant to GLP-1, compared to high-carbohydrate-no-fat (HFD) mice. Using 16S rDNA sequencing to assess the composition of the gut microbiota, the authors further found that in the ileum of HFD mice, F/B, Lactobacillus and Clostridium significantly increased, while Bacteroidota and Defferribacterota significantly decreased. Similarly, we observed the same changes in the microbiota of mice in the SE group (20). In addition, by comparing mice lacking microbiota (germ-free mice) with mice with normal microbiota showed that GLP-1 levels were higher in germ-free mice than in conventionally reared control mice (39). They added LCMUFA, which stimulates the production of short-chain fatty acids, to the diet of mice significantly improving levels of atherosclerotic lesions and inflammatory cytokine. In turn, these benefits were consistent with improvements in the gut microbiota, i.e., lower F/B ratios, increased Akkermansia abundance, SCFA-induced GLP-1 expression and increased serum GLP-1 levels. This highlights the critical role of dysbiosis in drug efficacy.

In addition, diet and drug injections may affect the microbiome, but how and to what extent they do so may vary. First, diet is important in shaping and influencing the gut microbiome. The composition and function of the microbiomes of both humans and other mammals are profoundly affected by diet. For example, high-fiber foods can increase the diversity of gut microbes, providing rich nutrients and fiber, which is conducive to the growth of gut microbes and the maintenance of diversity (40). Conversely, diets high in fat, sugar, and salt may lead to imbalances in the gut microbiome, increasing the risk of chronic inflammation and metabolism-related diseases. On the other hand, drug injections, especially the use of antibiotics, can also have a significant impact on the gut microbiota. The diversity and abundance of intestinal flora are reduced, which increases the risk of some diseases, such as enteritis and intestinal infection (40, 41). We found that the Sham group, that is, normal saline, did not directly impact on the intestinal flora of mice, and its intestinal flora structure was relatively stable compared with before. However, the use of drugs can destroy the intestinal bacterial structure of mice to varying degrees. The diversity of the bacterial community was reduced, and the abundance of conditional pathogens such as Clotridioides was enriched, In contrast, the abundance of some butyric-producing bacteria such as Lachnoclostridium and Rhodotella, was reduced. Of course, the effects of specific drugs on the intestinal microbiota structure of T2DM mice may vary depending on factors such as drug type, dose, duration of use, and the particular condition of the mice.

We analyzed the intestinal flora composition of mice in the experimental group by 16S rDNA sequencing. The abundance of Ruminococcaeae, Lachnospiraceae, Akkermansia and Verrucomicrobiaceae in different taxa in group IE increased. Microorganisms such as Ruminococcaeae and Lachnospiraceaede have been found to promote GLP-1 release from intestinal L cells (42–44). This is consistent with our funding. The results showed that intestinal flora rapidly and significantly affected L cells and GLP-1 content. The beneficial effects of the genus Akkermansia on systemic metabolism are caused by a bacterial protein belonging to the S41A family that improves glucose homeostasis and ameliorates metabolic disorders in mice primarily by stimulating thermogenesis and GLP-1 secretion *in vivo* (45). These data suggest that Akkermansia is closely associated with the development of T2DM. These findings provide new insights into the role of Akkermansia in the pathogenesis and pharmacologic mechanisms of T2DM. It is a common genus of bacteria that produces butyric acid and propionic acid for its anti-inflammatory effects (46, 47). This is consistent with our findings. Firmicutes and Bacteroides are the central intestinal microbiota *in vivo*, and the ratio of Firmicutes/Bacteroides is negatively correlated with glucose tolerance (48, 49); other studies have demonstrated that the ratio of F/B is higher in mice with T2DM and also in mice with obesity (50, 51). In addition, FBG and FINS were negatively related to Bacteroides and Akkermansia and positively associated with Lachnospiraceae_NK4A136_group, Odoribacter and Mucispirillum (51). Proteobacteria is a relatively abundant gram-negative bacterium in T2DM mice, and its increased abundance exacerbates the inflammatory response. In conclusion, the gut microbiota is another metabolically active region that affects drug safety and efficacy. In addition, the gut microbiota may be a good target for modulating or preventing drug-induced pharmacological or toxicological effects. Since the gut microbiota has an important metabolic role, it should be further studied shortly. Moreover, the reduction in liver steatosis observed in the FMT group may be attributed to the abundant Bacteroidetes found in the fecal donors from the IE group. The latest research suggested that the Bacteroidetes genera could improve metabolic disorders and alleviate non-alcoholic hepatic steatosis by activating the Bacteroidetes-folate-liver pathway.

Given the interactions between gut microbiota and diabetes drugs, there is growing awareness that altering microbiota composition can affect metabolic phenotypes and provide a rational basis for developing personalized treatments for T2DM that target gut microbiota (52, 53). FMT has been widely used recently and has become a recognized and popular way to treat disease (54). Although the body weight and blood glucose of SE group T2DM mice decreased after PEX168 intervention, HbA1C and HOMA-IR did not improve, but the improvement of blood glucose was further prompted after FMT treatment. Many studies have shown that the gut microbiota is closely related to glucose metabolism (55), insulin resistance (54), and insulin secretion (56). There is growing evidence that the gut microbiota plays a causal role in T2DM, Which has led to targeted therapies designed to alter microbiome composition (56). FMT is a method of treating disease by rebuilding the gut microbiota (57). FMT has consistently shown the ability to overcome dysbiosis by producing profound and lasting effects on the gut microbiota, which may be a new approach to treating T2DM (58). Wang et al. found that FMT improved insulin sensitivity, increased the diversity of intestinal microbial structures, and significantly increased the abundance of butyricogenes (24). At the same time, FMT treatment successfully reduced FBG and improved glucose tolerance in diabetic mice. Restore the balance of intestinal flora and promote host homeostasis (59). In one trial, obese individuals experienced positive changes in insulin sensitivity after receiving FMT treatment from lean, healthy individuals. Our study also found that after 8 weeks of FMT treatment, mice showed significant improvement in both peripheral insulin sensitivity and glucose tolerance. This is likely an increase in the abundance of beneficial bacteria. Our study, showed no apparent adverse reactions after FMT administration in mice; random blood glucose, fasting blood glucose, HbA1C, and HOMA-IR were all reduced, and no severe hypoglycemia occurred. FMT combined with PEX168 is superior to PEX168 alone in improving blood glucose control and insulin resistance, which provides a new direction for FMT to intervene in T2DM and FMT combined with hypoglycemic drugs to intervene in T2DM.

There are still limitations in this study remain. First, our experiments used only one animal model source, the T2DM model established by C57B/L mice, where the gut microbiota environment fed with the same diet is highly similar, and the gut microbiota may not be very different after drug intervention, assessing subtle differences or mechanisms by which the microbiota contributes to pharmacodynamic efficacy may not be sufficient. Secondly, the microbiome interacts with the drug, and the unsatisfactory effect in the drug may not be due to the difference of the microbiome, or the drug may change the microbial environment of the mouse itself, which further changes the metabolism and efficacy of the drug. Third, gavage may impact the intestinal mucosal barrier of mice, which can affect the structure of the gut microbiota and further influence the absorption and utilization of drugs. Finally, although we divided the FMT group and the Sham group and made a comparison, there was no fecal bacteria colonization analysis, and the experimental results may be biased.

## Conclusion

In summary, the data suggest that in T2DM mice, the antidiabetic drug PEX168 can effectively reduce body weight, improve insulin resistance, reduce inflammatory reactions, and prevent the development of diabetes. However, individual differentiation of intestinal flora can affect the efficacy of PEX168. Further studies showed that FMT treatment improved FBG, HBA1C and HoMA-IR in T2DM mice and further improved the efficacy of PEX168, demonstrating the importance of microbiome dysregulation in the pathogenesis of T2DM. Therefore, FMT may be considered as an effective method to enhance drug efficacy.

## Data Availability

The raw data supporting the conclusions of this article will be made available by the authors, without undue reservation.
